# Understanding the Metabolic Effects of Surgically Induced Renal Ischemia in Humans: A Temporal Approach

**DOI:** 10.3390/metabo15070462

**Published:** 2025-07-08

**Authors:** Bhargav Arimilli, Tyler A. On, Vaishnavi S. Srirama, Ye Yang, Gitanjali Asampille, Jeffrey R. Brender, Murali C. Krishna, Jessica Y. Hseuh, Viraj P. Chegu, Zachary Kozel, Sandeep Gurram, Mark W. Ball, William Marston Linehan, Daniel R. Crooks

**Affiliations:** 1 Urologic Oncology Branch, Center for Cancer Research, National Cancer Institute, Bethesda, MD 20892, USA; 2 Clinical Cancer Metabolism Facility, Center for Cancer Research, National Cancer Institute, Bethesda, MD 20892, USA; 3 Department of Internal Medicine, NYU Langone Health, New York, NY 10016, USA; 4 Radiation Biology Branch, Center for Cancer Research, National Cancer Institute, Bethesda, MD 20892, USA

**Keywords:** metabolomics, ischemia, NMR spectroscopy

## Abstract

**Background/Objectives**: Thousands of nephrectomies are performed annually in the United States, but the short-term metabolic effects of surgically induced renal ischemia remain unclear. The conventional metabolic markers used to characterize post-surgical renal function, such as creatinine and GFR, are measured in the serum but do not provide metabolic information about the renal parenchyma itself. We aimed to characterize the immediate metabolic effects of surgical ischemia on renal parenchyma within a temporal framework. **Methods**: Timed renal parenchyma biopsies were collected from eight patients undergoing nephrectomy for renal cell carcinoma both prior to and after ligation of the renal hilum. These samples were ground, extracted, and analyzed using nuclear magnetic resonance (NMR) spectroscopy to measure changes in lactate, succinate, glucose, alanine, and glycine levels. **Results**: Due to experimental limitations, we were only able to draw limited conclusions from three patients. Of the five remaining patients, all had significant increases in lactate and succinate levels as a function of time, though the degree to which these increases occurred varied between each patient. Glucose levels generally decreased in the renal parenchyma but did not necessarily correlate with lactate production, assuming all glucose underwent fermentation to lactate in a hypoxic environment. Alanine and glycine levels did not change in a predictable pattern across patients. **Conclusions**: There are significant changes in lactate, glucose and succinate levels within minutes of the onset of renal ischemia in human patients. The degree of change in the metabolites analyzed varied significantly between patients. The length of surgical ischemia must be considered during surgical procurement of tumor specimens for metabolomic analysis.

## 1. Introduction

The kidney is an exceptionally well-vascularized organ, receiving up to 25 percent of cardiac output [1]. The kidney exhibits a gradient of oxygenation, with the highest O_2_ levels present in the cortex and the lowest in the medulla [2,3]. As a result, specific regions of the kidney are differentially sensitive to changes in perfusion [4], though ultimately, prolonged ischemia-induced hypoxia affects the organ as a whole. Hypoxic changes to the renal parenchyma at the cellular level have been well documented. Early changes to isolated perfused rat renal proximal tubules subjected to hypoxia included both epithelial and endothelial cellular swelling [5]. Histopathologic analysis of ischemic porcine kidney demonstrated increasing tubular degeneration and inflammatory infiltrate with time, with biopsies collected at 10 min intervals post-clamp [6]. A study of renal ischemia–reperfusion injury in mice demonstrated significant ultrastructural changes including distended Bowman’s capsules and damaged proximal tubule brush borders [7]. In isolated rabbit proximal tubules, early ultrastructural changes during hypoxia included mitochondrial shrinkage, matrix condensation, and widened cristae [8].

Given the above-described ultrastructural changes to mitochondria during renal ischemia–hypoxia, the function of the electron transport chain (ETC) during ischemia is of interest due to its sensitivity to hypoxia. The major inputs to the ETC are electrons derived from the oxidation of NADH by complex I and from conversion of tricarboxylic acid (TCA) cycle metabolite succinate to fumarate catalyzed by complex II (succinate dehydrogenase; SDH), both resulting in the reduction of ubiquinone to ubiquinol. Mitochondrial complex IV catalyzes the terminal ETC transfer of four electrons to O_2_ to form two molecules of H_2_O. The proton motive force provided by complexes I, III, and IV facilitate ATP production in the mitochondrial matrix by complex V [9,10]. In the absence of O_2_ as an ETC substrate, the reactions catalyzed by complexes I and II are impaired due to the loss of electron flux. Thus, hypoxia leads to decreased capacity to form ATP via the ETC, and cells shift to anaerobic glycolysis and lactate fermentation to satisfy the need for cellular ATP regeneration. The result is an early and substantial surge in intracellular lactate as well as robust cellular lactate secretion, elevations of which can be observed systemically in the circulation during numerous disease states and mitochondrial disorders in humans.

There is a need for better understanding of the metabolic alterations that occur during surgically induced renal ischemia in humans. It is estimated that over 80,000 cases of renal cell carcinoma (RCC) were diagnosed in the United States in 2024, up from over 60,000 cases in 2017, an increase that can be attributed to better availability of computed tomography (CT) and magnetic resonance imaging (MRI) screening modalities [11,12]. At least 6–8% of all RCC cases arise due to hereditary acquisition of a genetic risk factor [13], and patients who carry renal cancer predisposition genes have an increased life-long risk of developing bilateral and/or multifocal renal tumors [14]. These hereditary RCC patients often need more than one renal surgery in their lifetime, and nephron-sparing partial nephrectomy often provides the best modality to manage recurrent and multifocal renal masses [15,16,17].

Clamping and unclamping of the renal artery is a routine component of partial nephrectomy. Given that ischemia–reperfusion injury remains the leading cause of acute renal failure, understanding the in vivo effects of surgically induced renal ischemia is of immense value [18,19]. Comorbidities may also render patients more vulnerable to post-surgical renal insufficiency. Diabetic nephropathy due to type II diabetes mellitus (T2DM) is the most common cause of chronic renal failure in the developed world, and hypertension (HTN) is both a cause and effect of chronic kidney disease and can have an additive pathological effect in conjunction with diabetic nephropathy [20]. Medications such as NSAIDs (Non-Steroidal Anti-inflammatory Drugs) and aminoglycoside antibiotics are widely prescribed but can cause kidney injury in certain patients [21,22].

A better understanding of the acute metabolic effects of surgical ischemia will also aid in the interpretation of metabolite levels measured in human tumor specimens. Even with operating room rapid tissue procurement procedures in place, the time between onset of tissue ischemia, resection, procurement and snap-freezing of tumor tissue can vary from seconds to hours. Similarly to the model presented by Keller et al. [23], we utilized a temporal framework in this study to explore the metabolic effects of surgical renal ischemia in a series of eight patients undergoing completion nephrectomies. We performed 1D ^1^H NMR analyses of renal parenchyma biopsies to evaluate temporal changes in the concentrations of glucose, lactate, succinate, alanine, and glycine. Our results demonstrate a clear impact of ischemia on the levels of glucose, lactate, and succinate in the human renal parenchyma, and they also highlight the substantial variability encountered from patient to patient.

## 2. Materials and Methods

### 2.1. Sample Procurement

Specimens were procured from eight patients from July 2022 to July 2024. Samples were obtained intraoperatively from patients undergoing surgery for renal cell carcinoma at the NIH Clinical Center (protocol NCI-97-C-0147). Intraoperative biopsies were taken both prior to renal artery ligation (“pre-ischemia” samples) to establish a metabolic baseline, as well as after ligation and induction of ischemia (“post-ischemia” samples). An 18-gauge needle biopsy gun was used for both pre- and post-ischemia samples. Following renal artery ligation, a timer was started, and post-ischemia samples were obtained at serial timepoints. A consistent time interval between biopsies was not used given the constraints of the surgical procedures and operating room environment, but all timepoints were carefully recorded. All biopsies were rapidly rinsed in saline, blotted dry on a paper towel, promptly frozen within one minute of biopsy in liquid nitrogen, and stored at −80 °C until grinding and extraction.

### 2.2. Sample Grinding

Each sample was weighed and then ground under liquid nitrogen using a Spex Model 6875 tissue grinder (Cole Parmer, Metuchen, NJ, USA). Each ground tissue sample was then quenched with 1 mL 50% methanol and transferred to a 15 mL conical vial containing 1.79 mL acetonitrile. An amount of 710 μL ddH_2_O was used to rinse and capture any remaining sample in each grinding tube, which was then transferred to the same 15 mL conical vial. The resulting mixture (2:1.5:1 ratio of acetonitrile–methanol–water) was stored at −80 °C for up to a week prior to extraction, using modifications to our published protocol [24].

### 2.3. Three-Phase Extraction

Each mixture was then extracted in chloroform according to our three-phase extraction protocol [24]. The upper polar phase was aliquoted and lyophilized overnight into several fractions. A fraction representing one quarter of the total lyophilized residue then underwent a precipitation step in 80% acetone to purify and remove any remaining contaminating protein. The purified residue was then reconstituted in 210 μL of a D_2_O-based NMR buffer (8.32 μM DSS-*d*_6_, 1 mM *d*_16_-EDTA, 2.9 mM K_2_HPO_4_, 2.1 mM KH_2_PO_4_, pD = 7.0), vortexed, centrifuged for 5 min at 21,000× *g*, and 200 μL of the supernatant was transferred to disposable 3.0 mm glass NMR tubes (Bruker Biospin, Inc., Billerica, MA, USA).

### 2.4. Protein Quantification Assay

A Thermo Scientific™ Pierce™ BCA protein assay kit (Thermo Fisher Scientific, Rockford, IL, USA) was used to quantify the amount of protein recovered from each sample. A standard curve was generated by serial dilutions of a stock bovine serum albumin solution. Each sample’s protein residue was dissolved and sonicated in protein extraction buffer (62.5 mM pH 7.5 Tris, 0.5% SDS, 1 mM DTT). Each resulting protein suspension was vortexed, placed on a heat block at 95 °C for 5 min, set on ice for 10 min, and then centrifuged at maximum speed for 10 min at 4 °C. The supernatant was diluted in a 1:10 ratio onto a 96-well microplate and 200 μL BCA working reagent was added and the plate sealed and incubated for 30 min at 37 °C. Finally, absorbance at 562 nm was measured using a Cytation 5 spectrophotometer. Each standard and sample were measured in triplicate. Samples with an absorbance value within 20% of the protein buffer’s absorbance value were excluded from further analysis.

### 2.5. NMR Analysis of Polar Extracts

NMR spectra were measured on a Bruker 16.4T, 700 MHz spectrometer (Bruker BioSpin GmbH, Rheinstetten, Germany) at 293 K. One-dimensional ^1^H spectra were collected with water suppression (ZGPR), a spectral width of 8196.7, an acquisition time of 2.05 s, and a recycle delay of 6 s, with 512 scans. All spectra were processed and analyzed using MestreNova software (version 14.2.1). ^1^H spectra were referenced to the DSS resonance at 0 ppm. Following spectral acquisition and analysis, the limit of detection (LOD) for each metabolite of interest was established by determining the smallest peak that could be integrated in the dataset. Metabolites of interest included lactate-3 (1.32 and 1.31 ppm; LOD not determined) succinate (2.40 ppm; LOD not determined), fumarate (6.51 ppm; LOD 0.03 nmol/mg protein), β-glucose (4.64, 4.63 ppm; LOD 0.40 nmol/mg protein), alanine (1.57, 1.56 ppm; LOD not determined), and glycine (3.54 ppm; LOD not determined). The area under the relevant peaks were quantified and referenced to the known amount of 1.585 nmol DSS present in 200 μL of the solvent buffer. The calculated amount of each metabolite was then normalized to the amount of protein recovered from each sample in the BCA quantification step.

### 2.6. Statistical Analysis

Paired *t*-tests were performed for every metabolite in each patient. Each group of replicate timepoints was compared to the corresponding baseline value. *p*-values resulting from these paired *t*-tests are reported under each patient’s metabolite curve in the Results section. A threshold of *p* < 0.05 was used to determine statistical significance. We were unable to perform statistical analysis on Patient 1, given that only one pre-ischemia sample was available for analysis. Using MetSizeR (version 2.2.0) [25], we confirmed that the sample size in our study was insufficient to perform any formal comparisons between sub-groups.

## 3. Results

**Figure 1:**
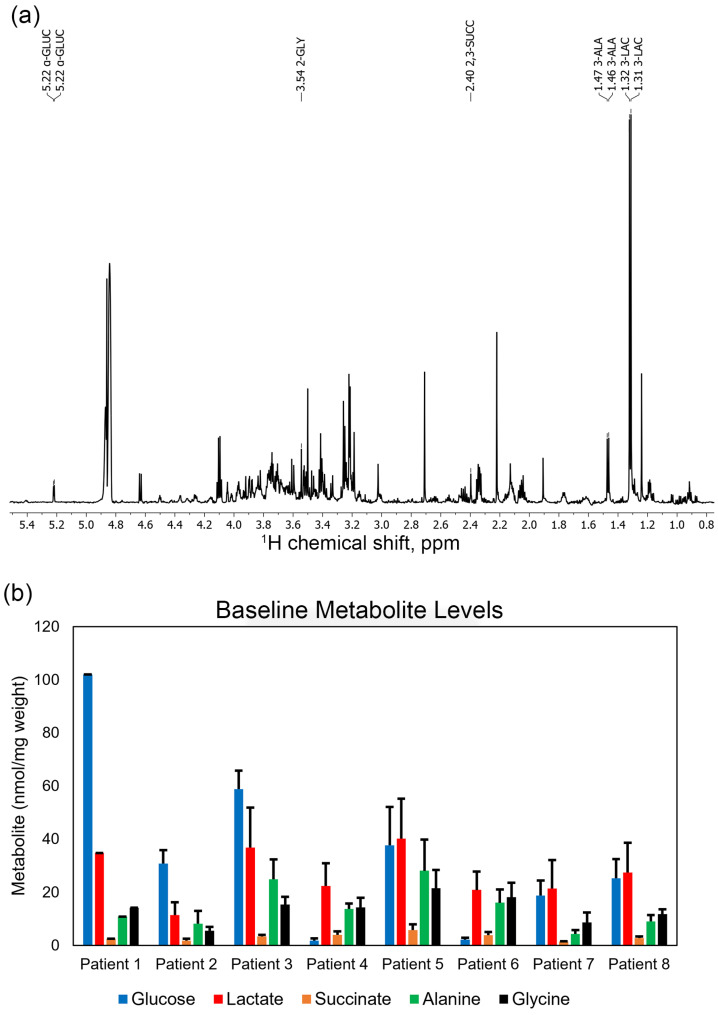
(**a**) 1D ^1^H NMR spectrum obtained from t = 48 min post-clamp renal parenchyma biopsy specimen of patient 1. 512 transients were recorded at 700.2 MHz with water suppression, a spectral width of 8196.7 Hz, acquisition time of 2.05 s, and additional relaxation delay of 6 s. The spectrum represents the polar metabolites extracted from 1.7 mg of tissue. Metabolites are as follows: 3-LAC; lactate, 3-ALA; alanine, 2,3-SUCC; succinate, 2-GLY; glycine; α-GLUC; alpha anomer of glucose. (**b**) Baseline metabolite levels in pre-ischemia biopsies obtained from each patient, normalized to extracted protein measured using the BCA assay.

The clinical characteristics of the eight patients from whom biopsies were obtained are shown in Table A1 (see Appendix A). All patients, with the exception of Patient 7, had a sporadic form of renal cell carcinoma. Patient 7 had RCC due to tuberous sclerosis (TSC). While eight patients were initially evaluated in this study, we ultimately focused our analysis on Patients 1–4 and Patient 8. Due to a limited number of data points, in-depth interpretation of the results obtained from Patients 5 and 6 was limited. Interpretation of Patient 7’s metabolic data was also limited because of unusual renal arterial anatomy that led to anastomotic blood flow during renal artery ligation. A typical ^1^H 1D NMR spectrum obtained from a renal parenchyma biopsy is shown in Figure 1a. Baseline renal parenchyma metabolite concentrations measured in biopsy specimens from each of the eight patients, obtained prior to renal artery ligation, are presented in Figure 1b.

### 3.1. Patient 1

Patient 1 was a 69-year-old male who underwent robotic radical nephrectomy of his right kidney for a 4.2 cm tumor with renal vein involvement. He had several co-morbidities, including HTN, T2DM, congestive heart failure, and stage III chronic kidney disease (CKD) likely resulting in chronic renal parenchymal vasculopathy. To our knowledge, he did not have any prior renal surgeries.

**Figure 2:**
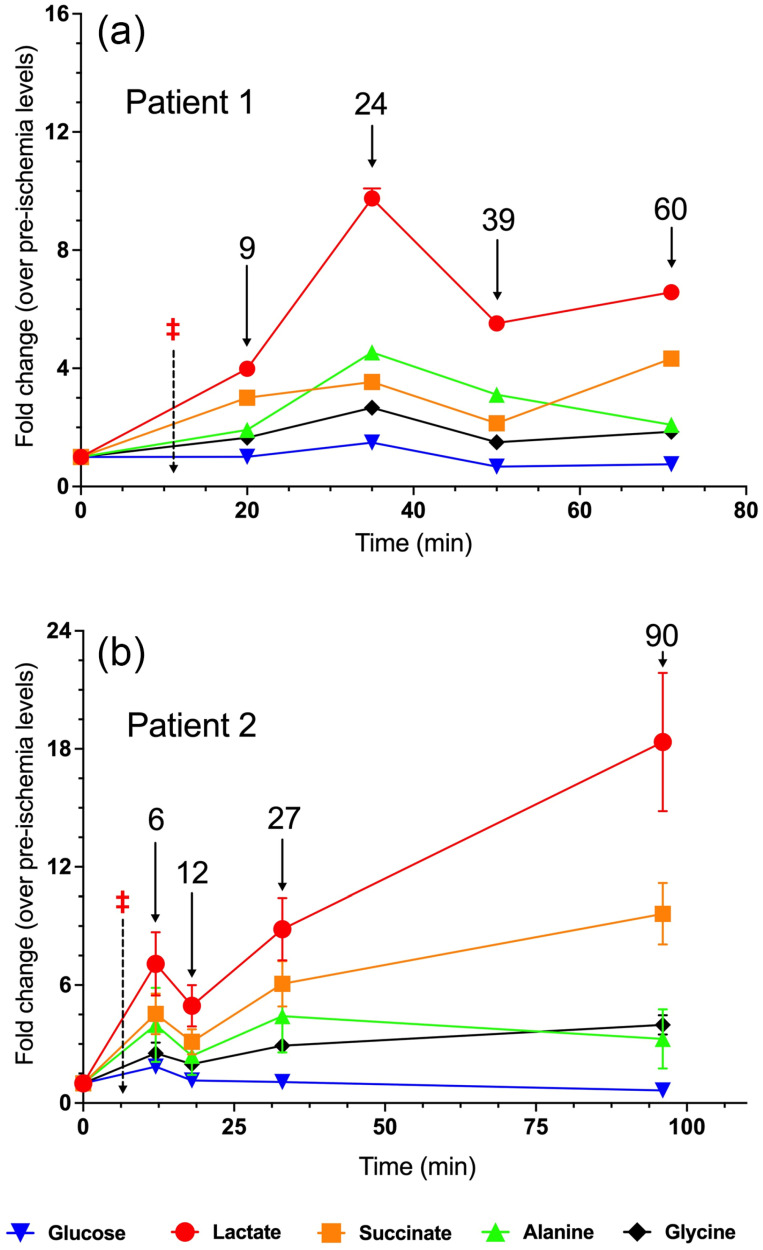
Fold change in metabolite levels in the renal parenchyma of (**a**) Patient 1 and (**b**) Patient 2. The annotated timepoints (solid arrows) refer to the number of minutes elapsed from renal artery clamping (denoted by ‡ and dotted arrow). The error bars in both plots represent standard deviation.

Patient 1’s metabolite trends are shown in Figure 2a. As expected, lactate initially increased with time, peaking at 24 min following arterial ligation before leveling off. Succinate levels rose to roughly four times pre-ischemia levels by the end of the time course. The trend for alanine levels mirrored those of lactate, albeit at a substantially smaller magnitude. Though glucose levels decreased slightly below pre-ischemia levels by the end of the time course, lactate levels rose out of proportion to glucose consumption (see Section 4). Finally, glycine levels remained relatively constant in comparison to other metabolites. Notably, Patient 1’s baseline renal cortex glucose concentration as shown in Figure 1 was remarkably higher than those in the rest of the cohort, nearly six times higher than the patient with the lowest baseline glucose. Surgical pathology reported a WHO Grade 3 clear cell RCC, as well as histological signs of chronic kidney disease with 20% of glomeruli globally sclerotic.

### 3.2. Patient 2

Patient 2 was an 88-year-old male who underwent robotic radical nephrectomy of his left kidney for a 7 cm renal tumor. Patient 2’s temporal metabolite trends are shown in Figure 2b. Lactate and succinate trended significantly upward, reaching a zenith of 18 times higher than baseline levels in the case of lactate. In comparison, Patient 1’s lactate reached a peak roughly 10-fold higher than baseline levels. Patient 2 had several comorbidities, including hypertension, hyperlipidemia, type II diabetes, and coronary artery disease which may have contributed to this large rise in renal parenchymal lactate levels following the onset of ischemia. Similarly to Patient 1, parenchymal glucose levels remained unchanged despite the increasing lactate levels. Alanine and glycine levels did not show a significant net change by the end of the time course. Surgical pathology indicated that the tumor was a WHO Grade 3 clear cell RCC.

### 3.3. Patient 3

**Figure 3:**
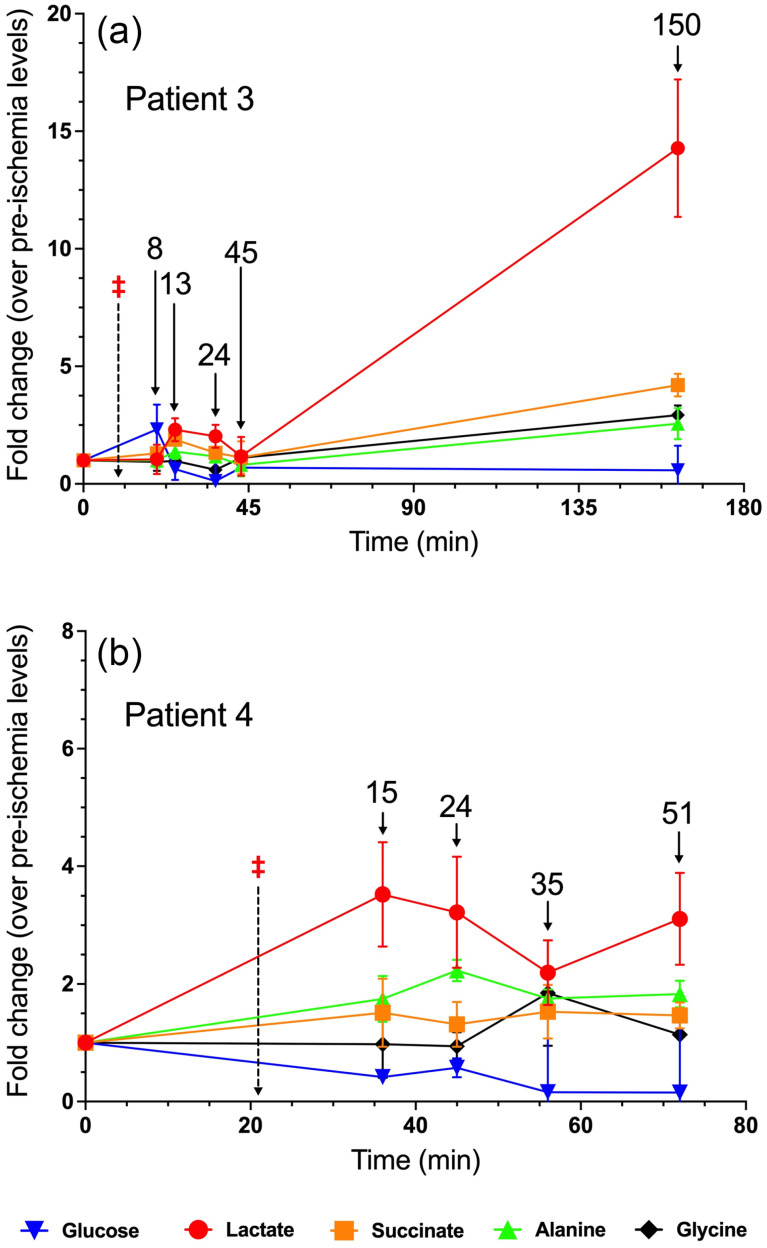
Fold change in metabolite levels in the renal parenchyma of (**a**) Patient 3 and (**b**) Patient 4. The annotated timepoints (solid arrows) refer to the number of minutes elapsed from renal artery clamping (denoted by ‡ and dashed arrow). The error bars in both plots represent standard deviation.

Patient 3 was a 76-year-old male who underwent robotic radical nephrectomy of his right kidney for a 6.5 cm tumor. He had a history of right bundle branch block (RBBB) and benign prostatic hyperplasia (BPH), as well as likely CKD. Patient 3’s lactate levels increased modestly at first, peaking at roughly twice pre-ischemia levels at t = 13 min, before decreasing back to rough baseline levels at t = 45 and then increased to nearly 15-fold at t = 150 min (Figure 3a). In contrast, while succinate levels initially followed the trend of lactate, succinate rose to only 4-fold higher than pre-ischemia at t = 150 min. There was also a modest increase in glycine and alanine levels at t = 150 min, whereas glucose levels at this timepoint were only slightly lower than pre-ischemia levels (Figure 3a). As seen in Patients 1 and 2, lactate rose well out of proportion to the small declines in glucose in the ischemic renal parenchyma. Surgical pathology indicated WHO Grade 2 clear cell RCC histology of the tumor, and the presence of several benign renal cysts.

### 3.4. Patient 4

Patient 4 was a 65-year-old male who underwent robotic radical nephrectomy of his right kidney for a 4.4 cm tumor. He had a history of hypertension (HTN), hyperlipidemia (HLD), and bladder cancer. The metabolite trends observed in Patient 4, particularly the change in lactate levels, were more modest relative to most other patients in this study (Figure 3b). For example, lactate peaked at under four times pre-ischemia levels, compared to nearly 18-fold in Patient 2. Succinate, unlike in most other patients, did not significantly increase during the time course (Figure 3b). Surgical pathology indicated a WHO Grade 3 clear cell RCC.

### 3.5. Patient 5

**Figure 4:**
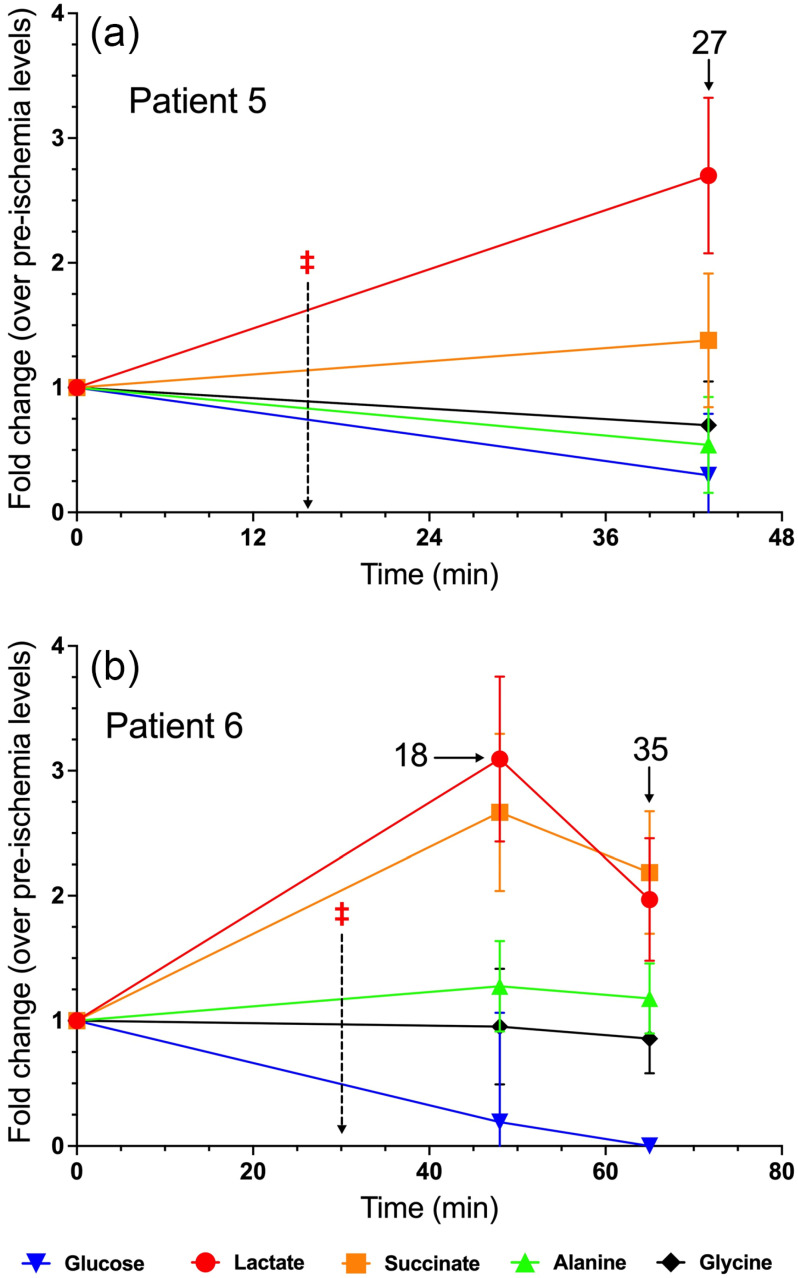
Fold change in metabolite levels in the renal parenchyma of (**a**) Patient 5 and (**b**) Patient 6. The annotated timepoints (solid arrows) refer to the number of minutes elapsed from renal artery ligation (denoted by ‡ and dashed arrow). The error bars in both plots represent standard deviation.

Patient 5 was an 81-year-old male who underwent robotic radical nephrectomy of his left kidney for a 2.5 cm tumor. He had a history of HTN and HLD. Only one post-ischemia timepoint was taken during this procedure, at 27 min post-clamp. Lactate levels were significantly elevated to 2.5-fold, while succinate levels increased non-significantly to 1.4-fold those of pre-ischemia levels (Figure 4a). In contrast, glucose levels declined significantly to 0.5-fold, and alanine and glycine levels declined, albeit non-significantly (Figure 4a). Surgical pathology identified the lesion as an oncocytoma. Given the patient’s advanced age and comorbidities, we opted to forgo repeated post-arterial ligation sample collections in order to not unduly expose the patient to extended time under anesthesia. Moreover, arterial calcifications were observed in this patient, including a substantial calcification at the origin of the left renal artery (Figure S2a).

### 3.6. Patient 6

Patient 6 was a 59-year-old female who underwent robotic radical nephrectomy of her left kidney with an 8 cm tumor, with retroperitoneal lymph node dissection. A renal biopsy indicated probable papillary RCC. She had a history of HTN, HLD, and hypothyroidism. Two post-clamp timepoints were available for analysis. Renal parenchymal lactate and succinate levels rose significantly to 3- and 2.5-fold, respectively, at 18 min post-clamp (Figure 4b). At t = 35 min post-clamp, lactate and succinate levels remained significantly elevated, though to a lesser extent than the previous timepoint (Figure 4b). Interestingly, glucose levels plummeted to 0.3-fold at t = 18 min post-clamp, and glucose plummeted to undetectable levels at t = 35 min (Figure 4b). Post-operative surgical pathology confirmed the diagnosis of papillary RCC, and notable dense chronic inflammation in the renal parenchyma.

### 3.7. Patient 7

Patient 7 was a 37-year-old male who underwent open radical nephrectomy of his left kidney for multiple solid and cystic renal masses, the largest of which were 3.0 and 2.2 cm. Notably, he had clinical manifestations of tuberous sclerosis, confirmed by a pathogenic germline mutation in the TSC1 gene. He had an extensive surgical history, including a prior right radical nephrectomy, two partial left nephrectomies, and multiple radiofrequency ablations for renal masses.

**Figure 5:**
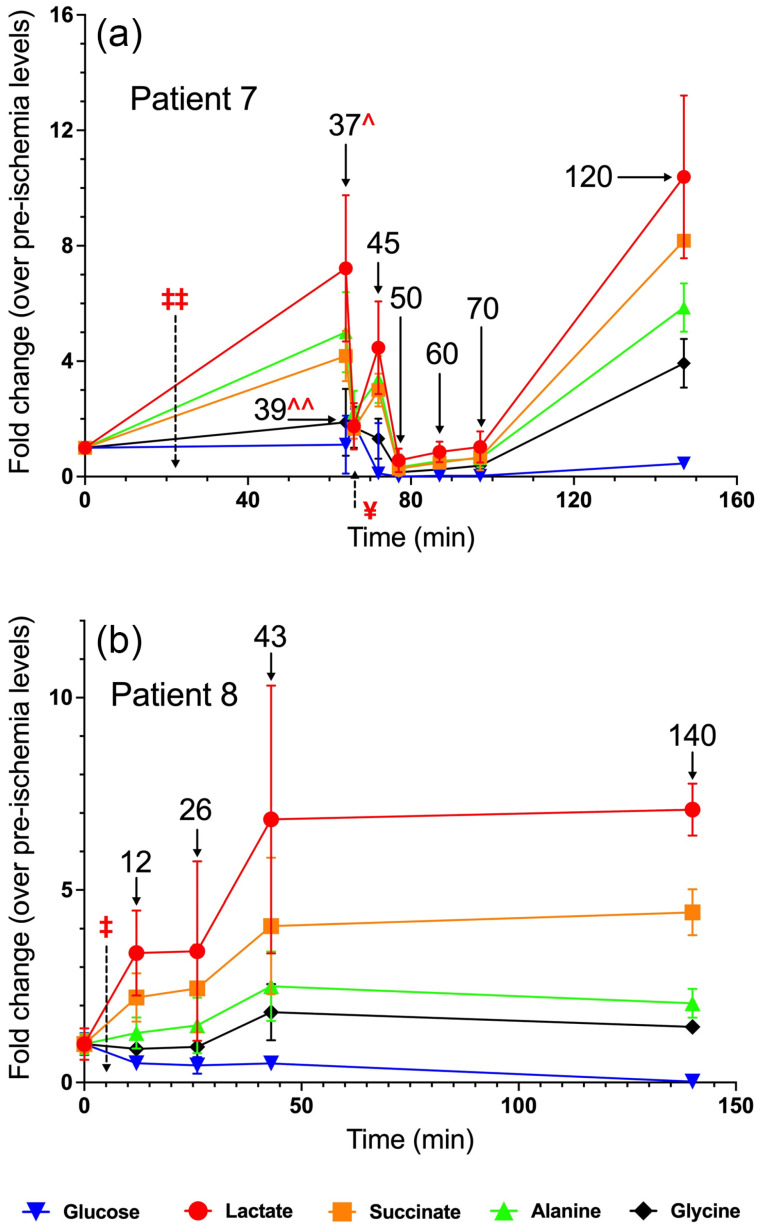
Fold change in metabolite levels in the renal parenchyma of (**a**) Patient 7 and (**b**) Patient 8. The annotated timepoints (solid arrows) refer to the number of minutes elapsed from renal artery clamping and ligation (denoted by ‡ and dashed arrow). In (**a**), ‡‡ refers to lower renal artery clamping, ¥ refers to hilar clamping, ^ refers to a lower midpole biopsy, and ^^ refers to a midpole biopsy. The error bars in both plots represent standard deviation.

Patient 7 had an unusual temporal metabolite profile, likely the result of both a complex renal arterial anatomy as well as his extensive prior surgical history involving the left kidney. The patient possessed two distinct renal arteries arising from the descending aorta (Figure S2b). During the present procedure, the lower renal artery was ligated 25 min after the pre-ischemia biopsies were taken (Figure 5a). Subsequently, the entirety of the renal circulation was occluded following ligation of the renal hilum 20 min later, at t = 45 min. Following the biopsy collected at t = 37, where lactate and succinate were increased albeit non-significantly, there was an abrupt decrease and fluctuation in metabolite levels evident in the samples collected from t = 39–60 min (Figure 5a). Lactate, alanine, succinate, and glycine levels were all significantly increased at the final timepoint, whereas glucose decreased significantly to 45% of control relative to pre-ischemia samples (Figure 5a). Chronic interstitial nephritis in the renal parenchyma of this patient was noted upon surgical pathology examination, and the kidney was found to harbor both multifocal clear cell RCCs and an oncocytic neoplasm.

### 3.8. Patient 8

Patient 8 was a 67-year-old male who underwent robotic radical left nephrectomy and retroperitoneal lymph node dissection for a 12 cm renal tumor. The renal artery was clamped and ligated shortly after obtaining pre-ischemia renal parenchyma biopsies, and post-clamp biopsies were taken at t = 12, 26, 43, and 140 min. Lactate and succinate levels in the renal parenchyma rose significantly to 3.4-fold and 2.2-fold at the first post-clamp timepoint (t = 12 min), and plateaued at ~7-fold and ~4-fold at t = 43–140 min post-clamp, respectively (Figure 5b). Glucose levels declined to about 50% of control from t = 12–43 min, and plummeted to just 3% of control at t = 140 min (Figure 5b). Surgical pathology indicated WHO Grade 3 clear cell RCC histology of the tumor.

### 3.9. The Role of Fumarate in Ischemia

**Figure 6:**
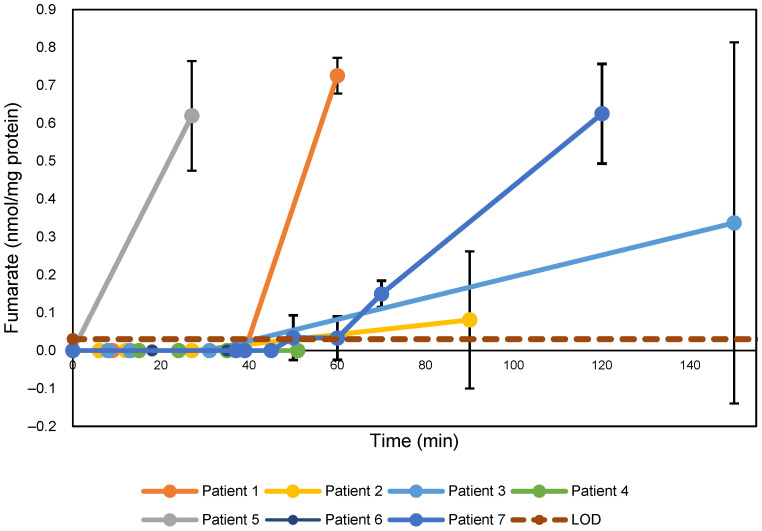
Fumarate levels in Patients 1–7; fumarate was not detected in Patient 8. The error bars represent standard deviation. The maroon dashed line refers to the limit of detection (“LOD”) for fumarate in our study, which was determined to be 0.03 nmol/mg protein.

Fumarate levels for all patients, reported in nmol/mg protein, are shown in Figure 6. Because fumarate was undetectable in all pre-ischemia samples, we were unable to calculate and report fold-change values for fumarate. We instead report these values in terms of concentration (nanomoles of fumarate per milligram of protein) as a function of time.

## 4. Discussion

The present work has demonstrated a clear temporal relationship between surgical ischemia and the levels of specific metabolites in the human renal parenchyma. To our knowledge, these data represent the first human study in which renal metabolite levels during surgically induced renal ischemia have been assessed in humans. We conclude that ischemia time must be considered during surgical procurement of tissue specimens. However, we also observed substantial patient-to-patient variability in both the initial metabolite levels in the renal parenchyma as well as the change in metabolite levels over time. These differences are likely due to the individual medical and surgical histories of each patient, as well as the unique anatomy of each patient and the variations in surgical approach employed during each procedure. These observations are important for the consideration of metabolite measurements made in human tumor specimens, where tumor ischemia time can range from minutes to hours depending on the complexity of the surgical procedure as well as logistical variations in intraoperative tissue procurement and processing procedures.

Baseline pre-ischemic renal parenchyma metabolite levels varied considerably between patients. For example, Patient 1’s baseline renal parenchyma glucose concentration was nearly six times higher than that of Patient 7. One possible explanation is Patient 1’s known history of T2DM. However, Patient 2 also had a known history of T2DM and had a baseline glucose that was lower than those of other patients who did not have known T2DM. Baseline renal lactate levels also varied widely within our patient cohort, perhaps reflecting different renal ischemic and surgical burdens to which these patients were previously exposed or differing capacity for renal intracellular lactate clearance via gluconeogenesis [26,27].

Our patients received between two and five liters of intravenous Lactated Ringers solution (LR) during their procedures (Table 1). LR is an isotonic crystalloid solution containing 28 mM sodium lactate whose osmolarity mimics that of human plasma though its electrolyte composition differs significantly. The impact of LR on plasma lactate levels has been evaluated previously in human patients, with one study reporting no significant difference in plasma lactate levels in healthy volunteers following administration of one liter of LR over the period of one hour, as compared to individuals infused with normal saline (NS) [28]. In another study, nearly twice the amount of LR (30 mg/kg) was administered to healthy volunteers via rapid bolus, and again no statistically significant difference in plasma lactate was observed [29]. While we cannot discount the influence of absorption and accumulation of lactate from LR infusions in the renal parenchyma of our patients, we consider it unlikely that intraoperative LR administration had a substantial impact on renal lactate levels in this study.

We observed ending lactate concentrations in the ischemic renal parenchyma [*lactate_final_*] that in most cases far exceeded that which would arise from complete fermentation of the free glucose initially present [*glucose_initial_*] in the non-ischemic tissue. Whereas the complete fermentation of one mole of glucose would yield two moles of lactate, we observed [*lactate_final_*/*glucose_initial_*] ratios in excess of 2:1, ranging from 2.2 to 37.8 (Table 1). Moreover, glucose levels in the ischemic renal parenchyma of most patients did not decrease to undetectable levels. If we assume that the ischemic kidney is a closed system following complete hilar ligation, our data suggest a source of glucose other than freely available cytosolic or extracellular glucose fueled the continued lactate production during ischemia.

Possible sources of additional substrates to fuel lactate accumulation in the ischemic renal parenchyma include gluconeogenesis [26,27,30], cytosolic pyruvate shunting via aminotransferases or other pathways [31], and glycogenolysis with subsequent anaerobic lactate fermentation. It is well-established that systemic glucose production from the human kidney occurs through gluconeogenesis [32,33,34], primarily occurring in the renal cortex, with circulating lactate as the predominant gluconeogenic precursor [27,35]. However, gluconeogenesis is an energetically costly process requiring hydrolysis of ATP and GTP and oxidation of NADH [31]. In contrast, hydrolysis of cytosolic glycogen stores for the fueling of glycolysis and lactate fermentation for cytosolic ATP production is a highly exergonic process which can occur in the absence of oxygen. Although renal glycogen stores are lower in abundance that those of liver or skeletal muscle, the mammalian kidney is not entirely devoid of glycogen [36,37]. In fact, renal glycogen levels were reported to decrease concomitantly with rises in lactate levels during experimentally induced ischemia in rats [38]. While the limited physical size of our human renal parenchyma biopsies precluded us from detecting glycogen resonances by ^1^H NMR, we propose that glycogenolysis coupled with anaerobic glycolytic fermentation fueled the continuing rise in renal lactate levels during ischemia.

We observed notable increases in succinate levels following induction of renal ischemia. Increased tissue succinate levels during hypoxia have been reported in classic studies of skeletal muscle [39,40], in cardiac muscle in various animals [41,42,43,44,45,46], and in hypoxic mouse kidney cells and tissues [8,47,48]. Historically, succinate accumulations in ischemia have been attributed to canonical, forward-turning TCA cycle activity [46,49]. Accumulation of the reduced ETC electron carrier and Complex II reaction product ubiquinol (UQH_2_), due to absence of the ETC terminal electron acceptor oxygen, results in succinate accumulation [46,50]. Re-oxidation of NADH produced by the α-ketoglutarate dehydrogenase complex and other TCA cycle reactions can be facilitated by mitochondrial transaminases and malate dehydrogenase [8,49].

An alternative source for ischemic succinate accumulation in mammals has also been explored, whereby the Complex II reaction runs in reverse as a fumarate reductase [45,47,51,52,53,54,55,56]. In microbes including E. coli and yeasts, fumarate reductase enzyme is encoded by genes that are distinct from those of Complex II and are activated during anaerobic conditions [57]. Nematodes, molluscs, and flatworms rely on fumarate reductase activity of Complex II for survival in low oxygen conditions [58,59]. The alternative electron carrier rhodoquinone is crucial for fumarate reductase activity in these lower organisms, as rhodoquinone possesses a reduction potential (E°) that is more negative than that of fumarate [59,60,61]. The extent to which mammalian Complex II exhibits fumarate reductase activity in vivo has been the subject of debate [45,46,50,55]. However, a recent study reported the discovery of rhodoquinone and fumarate reductase activity in mouse and human tissues [62]. Nevertheless, data from tracer studies of ischemia in cardiac and renal tissue in which the complete isotopologue distribution pattern is reported suggest that the majority of ischemic succinate accumulation derives from canonical TCA cycle flux with blockade of the forward reaction of Complex II [46,48]. For example, the m + 2 isotopologue of succinate increased in abundance in ischemic mouse kidneys relative to non-ischemic kidneys during an in vivo ^13^C_6_-glucose tracer experiment, whereas the m + 2 isotopologue of fumarate did not increase [48]. Furthermore, the ^13^C_6_-glucose-derived m + 2 isotopologue of succinate was far more abundant than the m + 3 succinate isotopologue in both ischemic and non-ischemic kidneys, suggesting that forward-turning TCA cycle glucose oxidation was responsible for the majority of succinate production in both conditions [63].

There are limitations to acknowledge in this study. First, our patient population was varied and the number of patients included in the study was insufficient for us to make population-level conclusions on the impact of renal ischemia on metabolite levels. Although great care was taken to obtain renal tissue biopsies as far away as possible from the tumor, we were unable to account for the potential impact of the presence of the renal tumor on metabolite levels in the renal parenchyma. Finally, for most patients, we were unable to record the time between renal artery ligation and complete ligation of the hilum (renal artery and vein), and we were thus unable to account for potential effects of venous backflow on renal perfusion.

Additional physiological parameters that cannot be practically controlled include the increased pressure of pneumoperitoneum during robotic surgery, increased pressure on the vena cava from surgical retractors during open surgery, and renal blood flow rate, which can be specific to the patient. Though no patients in this study had known renal artery stenosis or other renal hilar vascular malformations, variations in baseline arterial and venous blood flow could lead to inter-subject heterogeneity of venous backflow and clearance of certain metabolites, especially those that are in high flux such as lactate and glucose. Unlike the renal artery and vein which have clear and distinct times of ligation, it is more challenging to discern the extent of disruption to the renal lymphatic microcirculation during the dissection process. Therefore, it is challenging to quantify the effect that the renal lymphatic system, which mediates cortical interstitial fluid flow [64], has on the disposition of renal parenchymal metabolites at a given timepoint.

## 5. Conclusions

In this study, we present a novel, temporal approach to understanding the effects of surgical ischemia on the in vivo metabolic activity of the human renal parenchyma. In some patients there were increases in lactate and succinate levels within minutes of the onset of ischemia. Glucose levels decreased following the onset of ischemia in most patients, but the degree of glucose disappearance in some cases was far lower than that of lactate production. We speculate that glycogenolysis may have contributed to anaerobic lactate fermentation during renal ischemia in our patients. However, our inability to directly measure glycogen by NMR due to the small biopsy size precluded us from observing changes in renal glycogen levels over time. Future work using more sensitive means to measure changes in renal parenchymal glycogen concentrations during ischemia are merited. Finally, the inclusion of additional patients would have allowed for more formal statistical analyses and cohort-level observations. Nevertheless, our data demonstrate that tissue ischemia time must be considered during the procurement of normal and cancerous tissues for the purpose of metabolite measurements.

## Figures and Tables

**Table 1:** Ratio of the ending lactate concentration [*lactate_final_*] divided by the average pre-clamp glucose concentration [*glucose_intital_*] measured in the renal parenchyma of the patients. Final post-clamp timepoint is also listed for each patient.

Patient	[*lactate_final_*]/ [*glucose_initial_*]	Final Timepoint (Minutes)
Patient 1	2.2	60
Patient 2	6.8	90
Patient 3	8.9	150
Patient 4	37.8	51
Patient 5	2.9	27
Patient 6	18.7	35
Patient 7	11.9	120
Patient 8	7.7	140

## Data Availability

The raw data supporting the conclusions of this article will be made available by the authors on request.

## Supplementary Materials

The following supporting information can be downloaded at: https://www.mdpi.com/article/10.3390/metabo15070462/s1, Figure S1: Stacked 1D ^1^H NMR spectra from renal biopsies taken before (t = 0) and after ligation of the renal artery in Patient 1. A representative spectrum from each timepoint is shown, with spectral intensity normalized to the amount of protein present in each sample. Figure S2: Computed Tomography X-ray imaging of two patients in the study. (a) Coronal CT image without contrast demonstrating arterial calcification in Patient 5, including calcification at the takeoff (origin) of the left renal artery (black arrowhead). The tumor (white arrowhead) extended into the renal pelvis. (b) Arterial phase coronal CT of Patient 7 revealed two left renal arteries arising from the descending aorta (black arrowheads), which had to be clamped and ligated separately. The patient had undergone six nephron-sparing procedures in the left kidney prior to nephrectomy due to renal manifestations of tuberous sclerosis (TSC).

## Appendix A

**Table A1:** Patient characteristics at time of surgery.

Patient #	Genotype	Surgical Procedure	Tumor Size	Age at Time of Surgery	eGFR (Pre-Surgery)	Serum Creatinine (Pre-Surgery)	Intraoperative Medications	Prior Surgeries (Age at Procedure)	Co-Morbidities	Demographics
**1**	Sporadic	Robotic right radical nephrectomy	4.2 cm with renal vein involvement	69	53	1.43	LR—2.6 L 0.9% NaCl—900 mL Albumin 25%—100 mL Ephedrine—20 mg	Cholecystectomy (49Y) Appendectomy (55Y) Left inguinal hernia repair (61Y) Prostate biospy (69Y)	CKD (Stage 3) HTN T2DM GG2 prostate → benign bony lesions Afib CHF with EF 38%	Male White
**2**	Sporadic	Robotic left radical nephrectomy	7 cm	88	65	1.09	LR—2 L Albumin 25%—100 mL Ephedrine—20 mg Vasopressin—5 units	Cataract surgery (82Y) Renal biopsy (87Y) Percutaneous biopsy of liver lesion (88Y) Hemorrhoidectomy (88Y)	HTN HLD T2DM CAD/angina Macular degeneration	Male African American
**3**	Sporadic	Robotic left radical nephrectomy	6.5 cm	76	54	1.35	LR—4 L	Cholecystectomy (53Y) Umbilical hernia repair (73Y) Atrial appendage device placement (74Y) Afib ablation (75Y) Renal biopsy (76Y) Tonsillectomy Prostate vaporization	CKD RBBB BPH Afib	Male White
**4**	Sporadic	Robotic right radical nephrectomy	4.4 cm	65	103	0.69	LR—5 L Albumin 25%—200 mL Calcium chloride 10%—1000 mg Ephedrine—10 mg	Ureteroscopy/TUBRT (65Y) Renal biopsy (65Y)	HTN HLD Bladder cancer	Male White
**5**	Sporadic	Robotic left radical nephrectomy	2.5 cm	81	79	0.96	LR—3 L Ephedrine—20 mg Phenylephrine—250 mcg	2001, 2006: Prostate biopsy (59,65Y) Anal fistula repair (71Y) Left hand fracture repair with pins Tonsillectomy	HTN HLD Onychomycosis Seborrheic Dermatitis	Male White
**6**	Sporadic	Robotic left radical nephrectomy with retroperitoneal lymph node dissection	8 cm	59	80	0.84	LR—2 L Albumin 25%—100 mL Phenylephrine—80 mcg Hydrocortisone—25 mg	Left thoracentesis (58Y) Liver biopsy	HTN (off BP meds for 6 months) HLD Hypothyroidism	Female African American
**7**	Tuberous Sclerosis	Open left radical nephrectomy	Multiple solid and cystic renal masses—3.0 cm and 2.2 cm	37	75	1.23	LR—2 L	Right radical nephrectomy (20Y) Left kidney RFA ×3 (23Y) Left renal biopsy (29Y) Left robotic partial nephrectomy (31Y) Left open partial nephrectomy (33Y) Left ablation for presumed ccRCC (35Y) Wisdom tooth extraction	TSC-associated seizures Depression Anxiety	Male White
**8**	Sporadic	Robotic left radical nephrectomy with retroperitoneal lymph node dissection	12.2 cm	67	74	1.1	LR—3.5 L	none	HLD, HCM, CAD	Male, White

**Table A2:** *p*-values resulting from paired *t*-tests performed for each metabolite compared against baseline metabolite values in Patient 2. Statistically significant values are **bolded** with an asterisk (*).

Patient 2	Lactate	Succinate	Glucose	Alanine	Glycine
**t = 0**					
**t = 6**	0.051	0.075	0.100	0.126	0.120
**t = 12**	**0.010 ***	**0.022 ***	0.551	**0.034 ***	0.074
**t = 27**	**0.002 ***	0.054	0.639	0.067	**0.016 ***
**t = 90**	**0.006 ***	**0.001 ***	0.113	**0.049 ***	**0.008 ***

**Table A3:** *p*-values resulting from paired *t*-tests performed for each metabolite compared against baseline metabolite values in Patient 3. Statistically significant values are **bolded** with an asterisk (*).

Patient 3	Lactate	Succinate	Glucose	Alanine	Glycine
**t = 0**					
**t = 8**	0.894	0.094	0.310	0.999	0.740
**t = 13**	**0.005 ***	**0.001 ***	0.058	0.147	0.848
**t = 24**	**0.015 ***	0.058	**0.001 ***	0.398	**0.018 ***
**t = 31**	0.709	0.764	**0.010 ***	0.463	0.554
**t = 150**	**0.018 ***	**0.009 ***	0.197	**0.041 ***	**0.009 ***

**Table A4:** *p*-values resulting from paired *t*-tests performed for each metabolite compared against baseline metabolite values in Patient 4. Statistically significant values are **bolded** with an asterisk (*).

Patient 4	Lactate	Succinate	Glucose	Alanine	Glycine
**t = 0**					
**t = 15**	**0.007 ***	0.190	**0.038 ***	**0.033 ***	0.933
**t = 24**	**0.018 ***	0.285	0.098	**0.001 ***	0.780
**t = 35**	**0.018 ***	0.128	**0.013 ***	**0.003 ***	0.200
**t = 51**	**0.007 ***	0.103	**0.013 ***	**0.005 ***	0.481

**Table A5:** *p*-values resulting from paired *t*-tests performed for each metabolite compared against baseline metabolite values in Patient 5. Statistically significant values are **bolded** with an asterisk (*).

Patient 5	Lactate	Succinate	Glucose	Alanine	Glycine
**t = 0**					
**t = 27**	**0.011 ***	0.226	**0.020 ***	0.116	0.187

**Table A6:** *p*-values resulting from paired *t*-tests performed for each metabolite compared against baseline metabolite values in Patient 6. Statistically significant values are **bolded** with an asterisk (*).

Patient 6	Lactate	Succinate	Glucose	Alanine	Glycine
**t = 0**					
**t = 18**	**0.0002 ***	**0.001 ***	**0.00035 ***	0.174	0.816
**t = 35**	**0.003 ***	**0.001 ***	**0.00004 ***	0.323	0.399

**Table A7:** *p*-values resulting from paired *t*-tests performed for each metabolite compared against baseline metabolite values in Patient 7. Statistically significant values are **bolded** with an asterisk (*).

Patient 7	Lactate	Succinate	Glucose	Alanine	Glycine
**t = 0**					
**t = 37**	0.200	0.313	0.897	0.292	0.504
**t = 39**	0.264	0.210	0.197	0.253	0.288
**t = 45**	0.120	0.204	**0.025 ***	0.128	0.571
**t = 50**	0.228	**0.002 ***	**0.008 ***	**0.044 ***	**0.036 ***
**t = 60**	0.643	**0.007 ***	**0.009 ***	0.094	**0.048 ***
**t = 70**	0.935	0.234	**0.009 ***	0.174	0.080
**t = 120**	**0.008 ***	**0.003 ***	**0.049 ***	**0.011 ***	**0.006 ***

**Table A8:** *p*-values resulting from paired *t*-tests performed for each metabolite compared against baseline metabolite values in Patient 8. Statistically significant values are **bolded** with an asterisk (*).

Patient 8	Glucose	Lactate	Succinate	Alanine	Glycine
**t = 0**					
**t = 12**	**0.008 ***	**0.002 ***	**0.004 ***	0.220	0.300
**t = 26**	**0.008 ***	0.052	**0.024 ***	0.197	0.699
**t = 43**	**0.017 ***	**0.007 ***	**0.006 ***	**0.009 ***	**0.040 ***
**t = 140**	**0.000 ***	**0.000 ***	**0.000 ***	**0.002 ***	**0.005 ***

**Table A9:** Standard deviation for fold change calculations in baseline metabolite values for each patient. To calculate fold change in baseline metabolite values, the absolute value was divided by the average of all baseline values; the standard deviation of these quotients is reported in this table.

	Glucose	Lactate	Succinate	Fumarate	Alanine	Glycine
**Patient 1**						
**Patient 2**	0.16	0.42	0.38		0.60	0.26
**Patient 3**	0.12	0.41	0.17		0.30	0.20
**Patient 4**	0.45	0.38	0.31		0.15	0.25
**Patient 5**	0.25	0.22	0.23		0.45	0.17
**Patient 6**	0.32	0.33	0.30		0.31	0.24
**Patient 7**	0.30	0.50	0.08		0.33	0.43
**Patient 8**	0.29	0.41	0.23		0.27	0.15
