# Supplementary material for: Understanding the Metabolic Effects of Surgically Induced Renal Ischemia in Humans: A Temporal Approach
Source: Metabolites. 2025 Jul 8;15(7):462. doi: 10.3390/metabo15070462 (PMC12300224; doi:10.3390/metabo15070462)
Supplement: Supplementary file 1 [file metabolites-15-00462-s001.zip › metabolites-3661334-supplementary.pdf]

Supplementary Material

Understanding the Metabolic Effects of Surgically Induced Renal Ischemia in Humans: A Temporal Approach

Bhargav Arimilli[1,2,3], Tyler A. On[1,2], Vaishnavi S. Srirama[1,2], Ye Yang[1,2], Gitanjali Asampille[1,2], Jeffrey R. Brender[2,4], Murali C. Krishna[2,4], Jessica Y. Hseuh[1], Viraj P. Chegu[1,2], Zachary Kozel[1], Sandeep Gurram[1], Mark W. Ball[1], W. Marston Linehan[1,2*], Daniel R. Crooks[1,2*]

1 Urologic Oncology Branch, Center for Cancer Research, National Cancer Institute
2 Clinical Cancer Metabolism Facility, Center for Cancer Research, National Cancer Institute
3 Department of Internal Medicine, NYU Langone Health
4 Radiation Biology Branch, Center for Cancer Research, National Cancer Institute
* Authors to whom correspondence should be addressed DRC: crooksda@mail.nih.gov; +1-240-858-3700. WML: WML@nih.gov; +1-240-858-3700

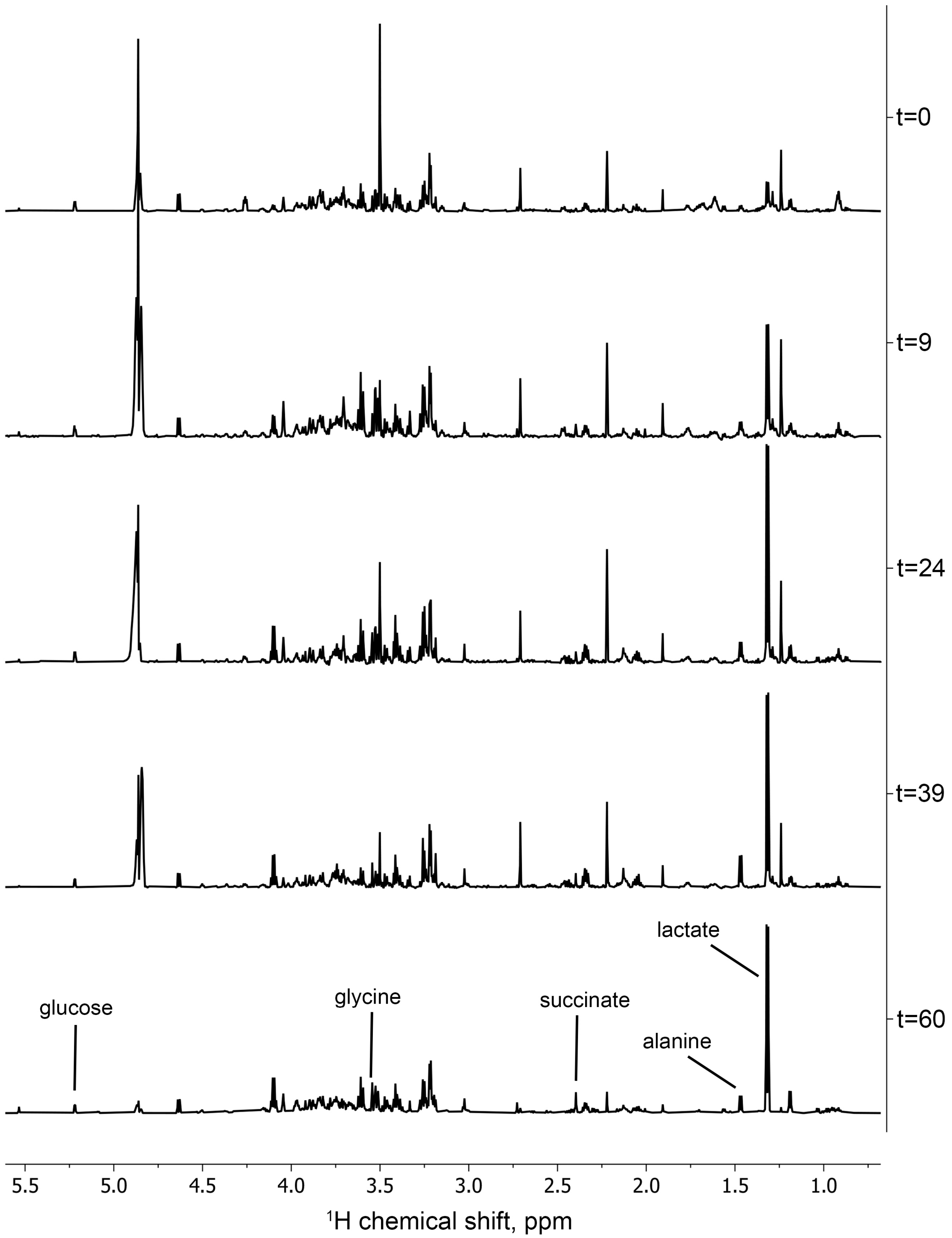

Figure S1: Stacked 1D ^{1}H NMR spectra from renal biopsies taken before (t=0) and after ligation of the renal artery in Patient 1. A representative spectrum from each time point is shown, with spectral intensity normalized to the amount of protein present in each sample.

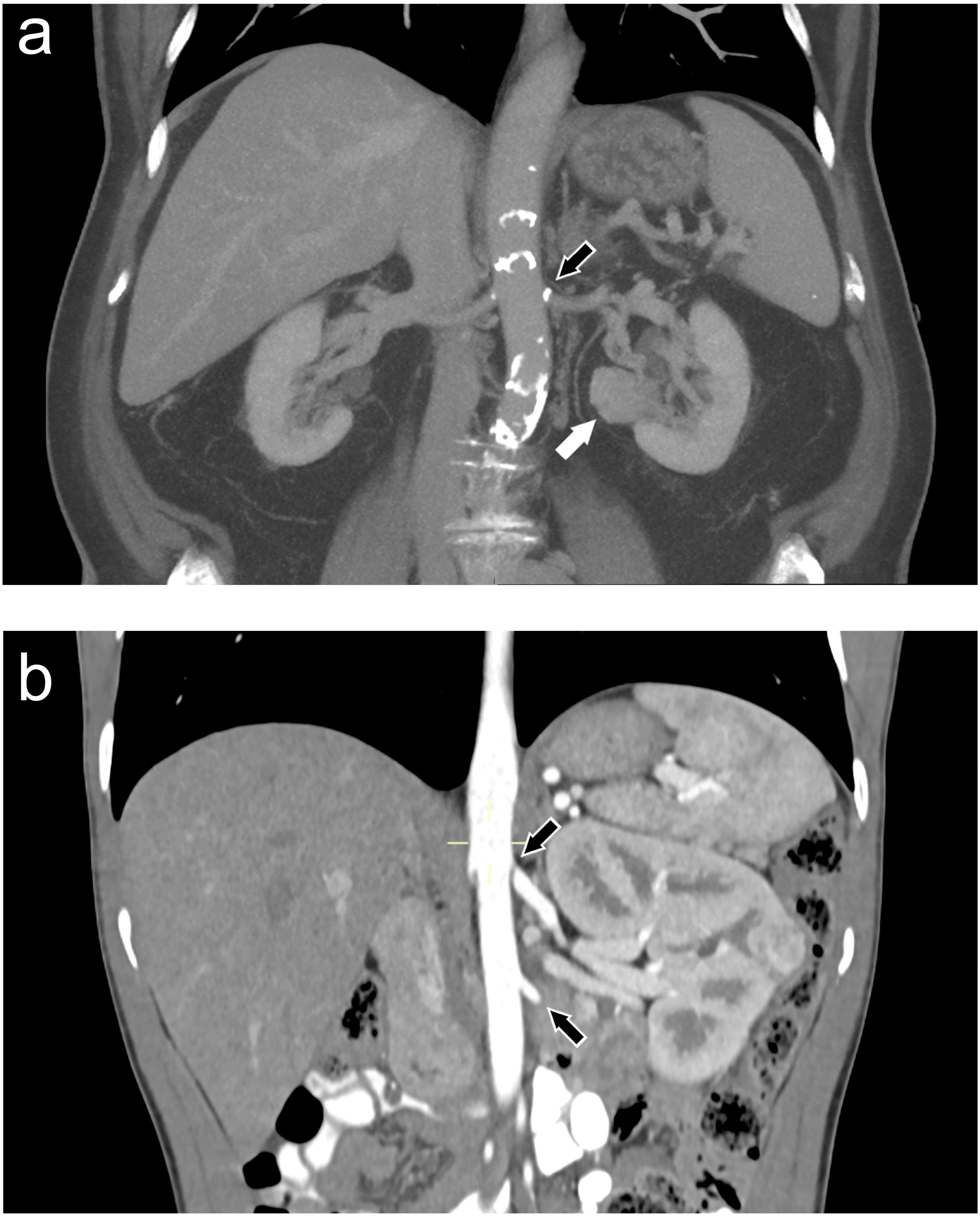

Figure S2: Computed Tomography X-ray imaging of two patients in the study. (a) Coronal CT image without contrast demonstrating arterial calcification in Patient 5, including calcification at the takeoff (origin) of the left renal artery (black arrowhead). The tumor (white arrowhead) extended into the renal pelvis. (b) Arterial phase coronal CT of patient 7 revealed two left renal arteries arising from the descending aorta (black arrowheads), which had to be clamped and ligated separately. The patient had undergone six nephron-sparing procedures in the left kidney prior to nephrectomy due to renal manifestations of tuberous sclerosis (TSC).
